# Weight Maintenance with Litramine (IQP-G-002AS): A 24-Week Double-Blind, Randomized, Placebo-Controlled Study

**DOI:** 10.1155/2015/953138

**Published:** 2015-09-07

**Authors:** Barbara Grube, Pee-Win Chong, Felix Alt, Ralf Uebelhack

**Affiliations:** ^1^Practice for General Medicine, Kurfürstendamm 157/158, 10709 Berlin, Germany; ^2^Zaluvida Corporate Sdn Bhd, E-16 Plaza Mont Kiara, 2 Jalan Kiara, 50480 Kuala Lumpur, Malaysia; ^3^Analyze & Realize GmbH, Waldseeweg 6, 13467 Berlin, Germany; ^4^Analyze & Realize GmbH, Weißenseer Weg 111, 10369 Berlin, Germany

## Abstract

*Background.* Litramine (IQP-G-002AS) was shown to be effective and safe for weight loss in overweight and obese subjects. However, long-term effectiveness on maintenance of body weight loss has yet to be ascertained.* Objective.* To assess effect of Litramine on maintenance of body weight loss.* Methods.* A double-blind, randomised, placebo-controlled trial on overweight and obese patients was conducted over two sites in Germany for 24 weeks. Subjects with documented previous weight loss of 3% over the last 3–6 months were randomised to groups given either Litramine (3 g/day) or a matching placebo. Primary endpoints were difference of mean body weight (kg) between baseline and end of study and maintenance of initially lost body weight in verum group, where maintenance is defined as ≤1% weight gain.* Results.* Subjects who were taking Litramine lost significantly more body weight compared to the subjects taking placebo who gained weight instead (−0.62 ± 1.55 kg versus 1.62 ± 1.48 kg, *p* < 0.001). More importantly, 92% of subjects in Litramine group were able to maintain their body weight after initial weight loss, versus 25% in placebo group. No serious adverse events were reported throughout.* Conclusion.* Litramine is effective and safe for long-term body weight maintenance.* Trial Registration.* This trial is registered with Clinicaltrials.gov identifier: NCT01505387.

## 1. Introduction

Globesity was a term coined by the World Health Organisation (WHO) to describe the escalating global epidemic of overweight and obesity (BMI ≥ 25 kg/m^2^). Since the 1980s, worldwide obesity nearly doubled, with the estimation that more than 10% of the world's population is now obese. Overweight and obesity have been associated with a high proportion of diabetes and ischaemic heart disease burden and also linked as a risk to certain cancers such as breast and colon cancer [1–3].

The increase in health risks results in higher burden on health care systems. In Europe, direct health care cost due to obesity is estimated to be approximately 7% of the total health care expenditure, which is comparable to cancer costs [4]. Similar cost is seen across the globe: in 2010, the estimated direct and indirect costs incurred as a result of obesity were in excess of USD 21 billion [5]. Global economic impact amounts to around 2.0% of global GDP (~USD 2 trillion), which was comparable to smoking, war, and terrorism [6].

Weight loss is clearly beneficial in terms of reducing morbidity risk. As little as 2% weight loss maintained in the long term will cut the risk of developing type 2 diabetes by 30% [7]. However, the key to these benefits is weight maintenance over long term instead of short-term weight loss and potential regain, which is the so-called “yo-yo effect.” A systematic review on weight loss and weight maintenance trials found that improvement in risk factors was only sustained if there was no weight regain after intervention, where even a slight regain had deleterious effects on the benefits obtained [8]. The cause of overweight and obesity is multifactorial, but a positive energy balance is often the main contributor. Consumption of food rich in fat and sugar, such as fast food, has rapidly increased over the years. The increased caloric intake has been shown to increase the risk of gaining weight, eventually leading to overweight and obesity [9, 10]. Therefore, treatments that aim at reducing calorie intake and absorption of dietary fat are effective approaches to reduce weight [11].

The investigational product, Litramine (IQP-G-002AS), is a patented natural fibre complex derived from* Opuntia ficus-indica*, which has been enriched with additional soluble fibre from* Acacia* sp. and coprocessed with cyclodextrin. The product is standardised for its lipophilic activity and therefore is able to bind to dietary fat and form fat-fibre complexes. The resulting fat-fibre complexes are not readily absorbed through the gastrointestinal tract and are eventually excreted through the faeces [12]. A previous randomised, placebo-controlled study has shown that the intake of Litramine successfully reduced body weight through the abovementioned mechanism [13]. However, the weight maintenance effect of Litramine has yet to be proven in clinical trial. Therefore, the objective of this current study is to determine the effect of Litramine on body weight maintenance after initial weight loss.

## 2. Method and Procedure

### 2.1. Trial Design

This double-blind, randomised, placebo-controlled study was conducted over 24 weeks (from January 2012 to September 2012) at two study sites in Germany. Germany has a significant population of overweight and obese subjects; 61.7% of German males and 45.2% of German females have a BMI ≥ 25 kg/m^2^ [14]. Subjects were randomly allocated to either the Litramine or the placebo group in a 1 : 1 ratio. Randomisation was done using a block size of 4 by an independent biostatistician using the randomisation scheme BiAS for Windows V9.2 (http://www.bias-online.de/).

All subjects gave written informed consent voluntarily. The clinical investigation was approved by the ethics committee of Charité-Universitätsmedizin Berlin and was performed in compliance with EN ISO 14155, the Declaration of Helsinki, and the Guideline for Good Clinical Practice (CPMP/ICH/135/95).

Eligible subjects included overweight and obese subjects (BMI 25–35 kg/m^2^), male and female, between the ages of 18 and 60 years, who had a documented weight loss of at least 3% over the last 3–6 months either from participation in weight loss clinical trials or weight loss regimens. Women of childbearing age were included but were required to use appropriate birth control methods during the duration of study. Subjects who had known sensitivity to the ingredients in the investigational product were excluded from the study. Other exclusion criteria included subjects with a history of medical disorders that may affect body weight (such as diabetes mellitus and Cushing's disease), presence of any active gastrointestinal (GI) disease or malabsorption disorders, history of GI surgery, history of eating disorders, history of cardiac disease or renal disease, pregnant and lactating women, subjects on medication that could influence GI function, and subjects using other antiobesity products.

At screening visit, inclusion and exclusion criteria were assessed. A detailed medical history was obtained from the subjects and a physical examination conducted by the investigator. Body weight and blood pressure measurement were also taken, along with blood samples for clinical chemistry, haematology, and lipid profiling.

Eligible subjects received either two tablets (1 g) of Litramine or matching placebo, to be taken three times a day with each main meal. The placebo tablet was identical in appearance to the Litramine tablet but contained 500 mg of microcrystalline cellulose as replacement for the active ingredient. Tablets were packed into bottles and labeled by an independent pharmacist for each subject according to the randomisation schedule. Both the investigators and the subjects were not informed of the treatment allocation throughout the study.

All subjects were encouraged to maintain a nutritionally balanced diet and to gradually increase their daily physical activity (30 mins of moderate intensity such as walking or cycling daily). However, no formal dietary restriction or any behavioral modification programmes were applied to simulate “free-living conditions.” The subjects were asked to keep a diary documenting their daily intake of the investigational product and also their calorie intake.

Subjects were followed up through scheduled visits with the investigators on week 12 and week 24, with telephone follow-ups in between visits on week 6 and week 18.

### 2.2. Endpoints

The primary endpoint of this study was to compare the difference in body weight (kg) at baseline and after 24 weeks of treatment with Litramine versus placebo in overweight and obese subjects. Body weight was measured using a calibrated weighing scale (Tanita BC-420 SMA; Tanita, Tokyo, Japan) in subjects wearing underwear and no shoes at screening, at baseline, and subsequently at 12-week intervals. A second primary endpoint looked at the maintenance of initial lost body weight (≤1% weight gain) in the Litramine versus placebo group.

Secondary endpoints included changes in mean waist and hip circumferences, body mass index (BMI), and mean body fat mass. Waist circumference (cm) was measured at the level midway between the lateral lower rib margin and the iliac crest while hip circumference (cm) was measured as the maximal circumference over the buttocks. Body fat content was measured by bioimpedance method using validated electronic weighing scales (Tanita BC-420 SMA). Subjects were also asked to complete an assessment on the feeling of satiety prior to the three main meals using a Control of Eating Questionnaire (COEQ) through a 4-point categorical scale (0 = no, 1 = slightly, 2 = moderate, and 3 = strong) [15]. Results were then compiled to measure changes in satiety levels at baseline and whilst taking Litramine.

### 2.3. Safety Parameters

Safety assessment included measurement of vital signs (such as resting blood pressure) and blood parameter investigations such as clinical chemistry (full blood count, liver and renal function), haematology, and lipid profile at 12-weekly intervals. Resting blood pressure was measured using standard devices. The subjects and investigators also subjectively evaluated the tolerability of the study medication at the end of the study. All adverse effects were recorded regardless of causality.

### 2.4. Statistical Analyses

The sample size for the primary endpoint of body weight loss effect was calculated based on the expected weight loss of the placebo and the control groups, according to the data from previous weight loss study [13]. Assumptions were made that placebo subjects gain at least 1.4 kg in mean body weight while Litramine subjects lose a moderate mean weight of 1.0 kg over 14 weeks. Based on two-sample *t*-test, a standard deviation of 2.7, a significance level of 5% (two-tailed), a power of 80%, and a 10% dropout rate, the calculated sample size was 25 subjects per group. This sample size was also found to be sufficient to show a difference in weight gain of at least 1.74 kg for second primary endpoint (body weight maintenance), with 1% (0.8 kg) of acceptable difference, considering a significance level of 5% (two-tailed) and a power of 80% (one-sample *t*-test with adaptation to nonparametric test). Sample size estimation was done using the software PASS V9.0 (Power Analysis and Sample Size, NCSS, Kaysville).

Demographic and baseline characteristics of efficacy and safety variables were assessed based on descriptive statistics. All primary and secondary variables, as well as safety variables, were evaluated as relative change within the groups. Repeated measures ANOVA was conducted to evaluate all efficacy variables using a repeated measure design.

For all statistical analyses, the level of significance (*p* < 0.05) was assumed. All values were presented as mean ± SD unless indicated otherwise. Efficacy and safety data were analyzed based on intention-to-treat (ITT) population, which was defined as subjects who have received at least one dose of investigational product. All statistical analyses were done using the SPSS Statistic software, V19.0 (SPSS, Chicago, IL).

## 3. Results

**Figure 1:**
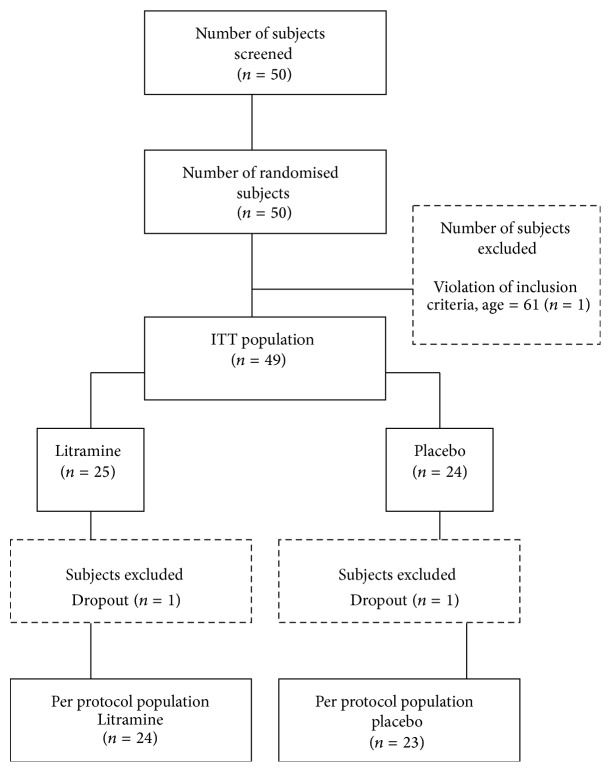
CONSORT diagram on study flow.

50 subjects were screened and subsequently randomised into two groups. The ITT population in this study consisted of 49 subjects; one subject was excluded due to the violation of inclusion criteria (Figure 1). In addition, 2 subjects had dropped out before the end of the study. For these subjects, measurements taken from the latest visit were used for the result analysis (last observation carried forward). Hence, the ITT analysis included 25 participants in Litramine group and 24 participants in placebo group.

The demographic characteristics of the ITT population are shown in Table 1. The baseline characteristics of Litramine and placebo groups were similar; there were no significant differences between groups in all parameters.

Table 2 shows the mean changes in primary and secondary parameters between baseline and week 24 for both groups. There was a statistically significant difference in mean body weight change between the Litramine and placebo groups at week 12: 0.24 (SD 1.10) kg versus −0.48 (SD 1.17) kg, respectively (*p* < 0.05). At the end of the study (week 24), subjects who were taking Litramine exhibited a mean weight loss of 0.62 (SD 1.55) kg compared to the placebo group who gained 1.62 (SD 1.48) kg instead (*p* < 0.001). Additionally, 92% of the subjects in the Litramine group showed body weight maintenance of baseline body weight compared to 25% in placebo group (*p* < 0.001).

Subjects who were on Litramine had little or no change in their BMI during the period of the study (−0.21 (SD 0.56) kg/m^2^). Conversely, those who were on placebo showed an increase in their BMI at the end of the study period (0.57 (SD 0.53) kg/m^2^). Litramine subjects also had significant reductions in waist and hip circumference compared to placebo (waist: −1.7 (SD 3.1) cm versus 0.7 (SD 1.5) cm; *p* < 0.001; hip: −0.6 (SD 2.9) cm versus 0.0 (SD 1.5) cm; *p* < 0.05).

Table 3 shows the mean changes in body fat mass and fat-free mass between baseline and week 24 for the Litramine and placebo groups. Only 47 subjects had their body fat mass analysed instead of 49. This was because two subjects did not have their body fat mass analysed at baseline; therefore, comparison of changes was not possible. Subjects in the Litramine group lost significantly more body fat (−1.0 (SD 1.7) kg) than the placebo group (−0.4 (SD 1.8) kg; *p* < 0.05), whereas fat-free mass loss was maintained. This indicates that Litramine induces weight loss through elimination of fat.

From the global assessment on the feeling of satiety, 60% of subjects who were on Litramine reported an increased feeling of satiety compared to 12.5% of subjects in the placebo group (Table 4).

Over the treatment period of 24 weeks, Litramine was well tolerated by the subjects. There were 16 adverse events reported by 13 subjects during the study period, 6 of which were from the Litramine group and 7 from the placebo group, but none were serious or considered to be related to the investigational product by the investigators. Clinical laboratory parameters did not show any significant changes or abnormalities during the study.

## 4. Discussion

In this study, we looked at the effect of Litramine on the maintenance of body weight after initial weight loss. According to the EU and US guidelines on the treatment of obesity [7, 11], one of the main criteria for treatment success is the maintenance of achieved weight loss. There are two main options for treatment of obesity: nonpharmacological and pharmacological. Nonpharmacological options are related to lifestyle and behaviour changes which include nutritional modification (e.g., decrease of calorie intake) and increase in physical activity (e.g., exercise). Pharmacological options are usually used as an adjunct to dietary measures and physical exercise. There are several therapeutic targets for obesity, such as appetite suppression (either through central pathways or through a physical action), interference with absorption of nutrient from the gastrointestinal tract, and increasing the body's basal metabolism rate.

Litramine acts via mechanical and physical modes within the gastrointestinal tract. Its ability to bind to dietary fats and prevent absorption, utilisation, and/or storage of the fat, resulting in weight loss, has been shown in previous studies on the product [12]. The current study showed that subjects on a prolonged (6 months) treatment with Litramine continue to experience a reduction in body weight, waist and hip circumferences, and body fat mass after their initial weight loss. It was also shown that the lean body mass is maintained during the study, thus indicating that weight loss by Litramine is mainly contributed by loss of body fat instead of other factors such as muscle loss or reduced water retention.

The current study also showed that those treated with Litramine experienced an increase in satiety feeling. Litramine contains soluble fibres which can gel and swell upon contact with water in the stomach. These effects increase the bulk volume and the viscosity in the gastrointestinal tract, which result in gastric distension, slowing down intestinal contractility and gastric emptying. Through this mechanism, soluble fibres can induce a feeling of satiety, reduce appetite, and possibly delay the onset of hunger [16, 17].

The current antiobesity drugs in the market often cause abdominal discomfort or act on the central nervous system and are associated with liver injury and cardiac and psychiatric side effects [18–20], leading to high dropout rates from drug clinical trials. As widely known, the lipase inhibitor Orlistat has side effects such as oily spotting which is often unacceptable for patients [18]. In more serious cases, anorectic drugs such as sibutramine and rimonabant have been withdrawn from the market due to their central nervous and cardiac side effect profiles which at times even resulted in fatalities [21–23]. In comparison, Litramine is an alternative option that is effective and has a good safety profile. No significant adverse effects were reported by the subjects in the current and previous studies of the product [12, 13]. Additionally, 95.7% of subjects in the current study rated the tolerability of Litramine as “good” or “very good,” which was identical to the rating for placebo. There was also a very low dropout rate which indicates good tolerability and acceptability.

### 4.1. Real Life Conditions

This study was conducted to simulate free-living conditions. The subjects were recommended to maintain a nutritionally balanced diet and not restricted in their food or calorie intake. Subjects were also not intensively scrutinised by the investigators. Over 24 weeks, 92% of the Litramine subjects successfully maintained their initial body weight loss, giving a positive indication that Litramine is effective and safe in long-term weight maintenance.

### 4.2. Study Limitations

The duration of the trial was limited to 24 weeks (i.e., six months) and no follow-up was performed after termination of the study. Many of the trials on weight maintenance were conducted for a longer period of time (12 months) [24]. Orlistat, for example, has been studied in one particular large multicentered weight maintenance trial which was conducted over two years [25]. However, it should be noted that the current study was carried out in accordance with the European Food Safety Authority (EFSA) guidelines, whereby a minimum of six months' evidence is required to prove weight maintenance [26]. Since there was no follow-up after termination of the study, any conclusions regarding the effect of Litramine on obesity-related diseases (e.g., diabetes, cardiovascular disease) cannot be made. Long-term studies would be required to study any effect on comorbidities such as the XENDOS trial which studied the use of Orlistat to prevent progression of diabetes in obese patients for over 4 years [27]. It would hence be favourable to conduct a long-term trial to study whether Litramine has a positive effect on obesity-related disease progression, such as diabetes and cardiovascular disease.

## 5. Conclusion and Implication

Evidence from this study and the other studies conducted [12, 13] supports the fact that Litramine should be considered as an efficacious means for weight management. Through the dual mechanisms of fat binding and increased satiety, Litramine can be used effectively alongside dietary and lifestyle modifications, with minimal side effects.

## Figures and Tables

**Table 1:** Baseline characteristics of intention-to-treat population. Values are presented as mean (standard deviation) unless otherwise specified.

Parameter	Intention-to-treat population (*n* = 49)		*p* value
	Litramine (*n* = 25)	Placebo (*n* = 24)	
Gender			
Men	6 (24%)	6 (25%)	1.000
Women	19 (76%)	18 (75%)	1.000
Age (years)	44.7 (11.4)	44.1 (11.2)	0.770
Body weight (kg)	78.2 (11.4)	79.3 (9.0)	0.516
BMI (kg/m^2^)	27.3 (2.1)	27.7 (2.4)	0.522
Waist circumference (cm)	98.8 (10.5)	100.3 (10.1)	0.785
Hip circumference (cm)	106.7 (7.9)	108.5 (8.4)	0.465
Body fat mass (kg)	26.8 (6.8)	28.6 (7.6)	0.400
Fat-free mass (kg)	51.3 (9.8)	49.9 (7.2)	0.996

**Table 2:** Mean changes in primary and secondary parameters between baseline and week 24.

	Litramine (*n* = 25) Mean (SD)	Placebo (*n* = 24) Mean (SD)	*p* value
Body weight (kg)	−0.62 (1.55)	1.62 (1.48)	<0.001
BMI (kg/m^2^)	−0.21 (0.56)	0.57 (0.53)	<0.001
Waist circumference (cm)	−1.7 (3.1)	0.7 (1.5)	<0.001
Hip circumference (cm)	−0.6 (2.9)	0.0 (1.5)	0.031

**Table 3:** Mean changes in body fat mass and fat-free mass in Litramine and placebo groups at week 24.

	Litramine (*n* = 25)	Placebo (*n* = 22)	*p* value
Body fat mass (kg)	−1.0 (1.7)	0.4 (1.8)	0.014
Fat-free mass (kg)	−0.4 (1.4)	1.2 (1.5)	0.024

**Table 4:** Change in feeling of satiety in Litramine and placebo groups between baseline and week 24.

	Litramine group		Placebo group	
Satiety	(*N* = 25)		(*N* = 24)	
	Number	%	Number	%
Decreased	1	4.0%	10	41.7%
Unchanged	9	36.0%	11	45.8%
Increased	15	60.0%	3	12.5%
*p* value	<0.001		0.057	
